# Tetrode Recording from the Hippocampus of Behaving Mice Coupled with Four-Point-Irradiation Closed-Loop Optogenetics: A Technique to Study the Contribution of Hippocampal SWR Events to Learning

**DOI:** 10.1523/ENEURO.0087-18.2018

**Published:** 2018-09-04

**Authors:** Dámaris K. Rangel Guerrero, James G. Donnett, Jozsef Csicsvari, Krisztián A. Kovács

**Affiliations:** 1 Institute of Science and Technology, Klosterneuburg 3400, Austria; 2 Axona Ltd, St Albans AL3 6PA, United Kingdom

**Keywords:** hippocampus, learning, memory, multi-unit recording, optogenetics, sharp-wave ripple

## Abstract

With the advent of optogenetics, it became possible to change the activity of a targeted population of neurons in a temporally controlled manner. To combine the advantages of 60-channel *in vivo* tetrode recording and laser-based optogenetics, we have developed a closed-loop recording system that allows for the actual electrophysiological signal to be used as a trigger for the laser light mediating the optogenetic intervention. We have optimized the weight, size, and shape of the corresponding implant to make it compatible with the size, force, and movements of a behaving mouse, and we have shown that the system can efficiently block sharp wave ripple (SWR) events using those events themselves as a trigger. To demonstrate the full potential of the optogenetic recording system we present a pilot study addressing the contribution of SWR events to learning in a complex behavioral task.

## Significance Statement

Our custom optogenetic microdrive design takes into consideration the small size and force of the mice and offers an unusually high number of channels to collect murine electrophysiological data. The laser delivery is optimized to irradiate the brain at four points so targeting structures as large as the dorsal CA1 region becomes possible. The described technique provides a cutting-edge tool to study several aspects of cognition, learning, memory, sleep, and decision making. All types of experiments where a larger brain area needs to be illuminated during extracellular recordings could exploit our newly developed implant. The preliminary data on the effect of sharp wave ripple (SWR)-blockade, presented here as an illustration of the new method, could inspire additional experiments using larger number of animals.

## Introduction

In the present work we developed a new type of implant for mice to test whether optogenetic inhibition of sharp wave ripples (SWRs) by affecting a large population of principal cells in the dorsal CA1 can cause impairment in the recall of recently learned spatial memories. To better understand the changes of hippocampal assemblies during learning, quiet immobility, and in the subsequent recall stages, we also needed to implement the possibility of massively parallel extracellular recording. We therefore developed a novel microdrive array incorporating four optic fibers targeting the dorsal CA1 areas bilaterally and capable of moving independently eight electrode bundles (15 tetrodes, 60 channels in total). To verify the suitability of the new implant and at the same time collect a preliminary dataset on our scientific question we opted to use a goal learning task on the cheeseboard maze because past work showed that the reactivation content of SWRs following this task could specifically predict the future behavioral performance of the animal (Dupret et al., 2010), while the range and complexity of the free movements required for this task exploited the features of the new implant to the largest extent possible.

During sleep and quiet rest, patterns neuronal activity of the previous active waking periods are reactivated, and this phenomenon is prominent during SWR events (Wilson and McNaughton, 1994). This reactivation has been suggested to promote memory consolidation by replaying memory traces. Inspired by this concept, many studies addressed the question of whether SWR during sleep and quiet rest periods (sSWR) are needed for learning and the stabilization of new memories. The first experiments electrically stimulated the ventral hippocampal commissure to disrupt sSWRs (Ego-Stengel and Wilson, 2009; Girardeau et al., 2009) and led to delayed learning in two different spatial learning paradigms that spanned several days. Subsequently, awake SWR events (Jadhav et al., 2012) were also disrupted similarly in rats performing a combined working and reference memory task. Disruption of waking SWRs did not lead to reference memory deficit, however, it impaired the working memory performance of the animal. One drawback of these experiments has been that such electrical stimulation is not cell-type specific, and can cause elevation of intracellular Ca^2+^ levels (Zugaro et al., 2005) that can lead to unwanted synaptic plasticity, at least in some cells. Because of this potential limitation, more recent studies examining the role of SWRs in the stability of place fields used optogenetic silencing. Our first study relying on optogenetic disruption of sSWRs did not point to an effect on place field stability: after exposure to novel environments, sSWR silencing did not interfere with the formation of stable CA1 place cell maps (Kovács et al., 2016). Contrarily, another study saw a destabilization of selected novel place field assemblies following a similar optogenetic sSWR blockade (van de Ven et al., 2016). More recently, hippocampal place cells were optogenetically inhibited during waking SWRs at reward locations during a goal-learning task on a cheeseboard maze. However, in this work the optogenetic intervention was deliberately local, influencing only a small proportion of hippocampal cells and non-inhibited cells were used as a control population (Roux et al., 2017). Because of the local light application, no behavioral impairment was seen there, but the manipulation destabilized the place fields in the inhibited cells. Therefore, so far, spatial learning impairment has only been demonstrated through electrical stimulation-mediated SWR disruption.

## Materials and Methods

### Animals

Three C57/BL6 female mice expressing an archaerhodopsin-EGFP were used in the present work. The animals were obtained from intercrosses between two established mouse lines, the Tg29 strain (JAX 005359) and the Ai35 strain (JAX 012735) that were purchased from The Jackson Laboratory under the indicated numbers. Further information can be found in our previous publication using the same intercross to produce optogenetically responsive animals (Kovács et al., 2016). All procedures involving experimental animals were conducted in accordance with the Austrian federal Law for experiments with animals and under a project license approved by the Austrian Federal Ministry of Science, Research and Economics (project license number: BMWFW-66.018/0015-WF/V/3b/2014).

### Optic ferrules and fibers

Optic ferrules coupled to high numerical aperture poly-methyl-methacrylate (PMMA) optic fibers were ordered from Doric Lenses (catalog number MFC_240/250-0.63_15mm_MF1.25(GK)_C45) and processed in the same manner as in Kovács et al. (2016).

### Microdrive building

Given the novelty of the technique, a detailed description of microdrive building is provided in Results. Tetrodes used in the microdrive were of tungsten wire covered by H-Formvar insulation covered with butyral bond coat (California Fine Wire). The contactor pins were from Mill-max (Interconnects series 861 gold pins). The parts of the microdrive as conceived and designed in the present work can be ordered from Axona Ltd.

### Surgery

The procedure was conducted in compliance with the applicable laws and guidelines of Austria and of the European Union.

Animals were initially anesthetized with 5% isoflurane, shaved, and then injected with 0.1 mg/kg buprenorphine and 10 mg/kg baytril. After shaving and disinfection, eyegel (GenTeal, Thea Pharma GmbH) was distributed over the eyeballs to avoid dehydration. During further steps of the surgery, isofluorane was set back to 1% in 1 dm^3^/min oxygen flow, and every 120 min, 0.1 ml of 5% glucose was given in physiologic solution to the animal in the form of an intraperitoneal injection. An initial incision was made with a scalpel-blade from between the eyes and, to avoid hurting the neck muscle, the opening was completed with scissors at the occipital pole. The skin, together with the neck muscle, was held apart with surgical thread (VICRYL; Ethicon) instead of using clamps, and was moistened regularly with physiologic solution. The skull bones were thoroughly dried and cleaned, the desired edges for bilateral craniotomy were marked by superficial drilling with drillbits of 0.5 mm (Hager and Meisinger GmbH, 1RF 005), and the following microscrews (M1.0) were implanted after predrilling with drillbits of 0.7 mm (Hager and Meisinger GmbH, 1RF 007): two in each os frontale, two in each os parietale (laterally from the planned craniotomy), and two in each os occipitale. The latter ones were targeted to touch the surface of the cerebellum and served as ground and reference electrodes. Subsequently a first layer of dental cement was used to establish contact between the skull bone and the screws, and then the craniotomy was completed above the right (centered at AP = 2.08; ML = 1.65) and left (centered at AP = -2.08; ML = -1.65) dorsal portion of the hippocampus. Using fine tipped forceps, the dura mater was removed together with the rectangular piece of skull bone, subsequently the microdrive was positioned over the craniotomy. Before the start of the surgery, the electrodes were moved to close to their uppermost position (tetrode tips located 150 µm higher than the conical tips of the optic fibers), and during implantation the optic fiber tips served as reference: from the point of their touching the brain surface, they were lowered down to a depth of exactly 1000 µm (upper half of the stratum oriens) in steps of 250 µm after touching the brain surface. A pause of 90 s was kept between each lowering step. After implantation, the edge of the bottom part of the drive was connected with the previously built cement wall using dental cement except on one side, after which the cannulae, the tubing of the optic fibers and the exposed neocortex were sealed with paraffin wax that is very soft at 37°C (although not liquid) and the elctrodes (or, if placed somewhat higher, rather the steel cannulae holding them) can freely move through it, either upwards or downwards. Finally, the cement wall was closed. The wires from the occipital screws were soldered to the ground wires at the occipital surface of the drive already connected to the five ground pins. To close the wound, the frontal skin was stitched together using the same piece of surgical thread that held the skin apart, while the edge of the lateral and occipital skin was glued to the dental cement wall. A typical surgery lasted 6 h. During a one-week-long postoperative recovery period, Metacam (1.5 mg/ml meloxicam suspension, Boehringer-Ingelheim) was given in the drink and the skin around the implant was disinfected daily with Betain (10% povidone-iodine) and local anesthesia with Xylocain (2% lidocaine-hydrochloride, Astra-Zeneca) was also delivered if needed. During the second week after surgery, the tetrodes were lowered into the CA1 regions of the hippocampus (to the stratum pyramidale) over a further period of around 4–6 d.

### Recording and analysis of the electrophysiological signal

Wide-band (0.4–9 kHz) recordings of local field potentials (LFPs) and multi-unit activity were amplified 1000-fold via a 64-channel dacqUSB amplifier (Axona Ltd., St. Albans) and digitized at 24 kHz using a 64-channel analog-to-digital converter built into the dacqUSB system unit (Axona). To reduce cable movement artifacts and to reduce the ratio of the noise recorded by the cable, a 60-channel unity-gain preamplifier headstage was designed in-house by wiring together three printed circuit boards that were driven by 5 V. The contactor pins of the headstage preamplifier were from Mill-max (Interconnects series 860 male/male pins), and the layout of the contactor pins matched the 5 × 13 layout of the microdrive lid. The headstage also had 5-V sockets for two LED panels that were plugged in during the exploration sessions to enable 2 point tracking, the position of the animal could thus be continuously registered using an overhead camera and the inbuilt tracking function of the dacqUSB SYSTEM UNIT in 2 point mode. In addition to the tracking, all cheeseboard sessions were videotaped.

To isolate single units (i.e., putative neurons), the data were processed and analyzed off-line. First, action potentials were extracted from the raw recordings by computing the power in the 800- to 9000-Hz range using a sliding window (12.8 ms). Subsequently, all the action potentials with a power of >5 SD from the baseline mean were selected and their features were defined in a 16-dimensional space using principal component analysis. The action potentials were subsequently segregated along their features into clusters that corresponded to putative single units (Harris et al., 2000) using KlustaKwik 3.0 (http://klustakwik.sourceforge.net/). Finally, such clusters were split, merged, and refined manually using Klusters (http://neurosuite.sourceforge.net/) to obtain units with clean refractory periods in their temporal autocorrelograms. Only units independent of each other and showing stable activity throughout the whole 9-h-long recording were included. Pyramidal cells and interneurons in the CA1 region were discriminated by their autocorrelograms, overall firing rate, and waveforms. The efficiency of the optogenetic inhibition was verified by measuring the decrease in the activity of individual pyramidal cells during regularly delivered 500 ms-long laser pulses in the course of the last 30-min-long sleep/rest the same way as in Kovács et al. (2016). For statistics, the STAT 5.4 UNIX software package 3 was used.

In total, 60 putative pyramidal cells could be isolated from three animals in the SWR blockade condition, while 76 putative pyramidal cells from the same three animals in the control condition.

### Ripple detection and optogenetic intervention

The on-line ripple detection was done the same way as described in Kovács et al. (2016), the wiring diagram of the analog ripple detector device can be found in Nokia et al. (2012). Briefly, the said detector emitted a 200-ms-long TTL signal, which was either delayed by 1.32 s (control) or not (sSWRs blockade), and was then fed into a COBOLT Jive 300 mW DPSS green (561 nm) laser manufactured by Omicron. The light was guided into a Ø1500 μm mother cable that in turn channeled it into four Ø200 μm daughter cables via a low-friction rotatory joint (Prizmatix).

### Behavior

Before implantation, animals were habituated to the cheeseboard and pretrained to become familiar with the task. Similarly to the rat cheeseboard used in our laboratory, the mouse cheeseboard had a diameter of 120 cm, had 177 wells placed according to the layout visible in Figure 5, and there was a distance of 5 cm between the neighboring wells. However, wells were fully cylindrical and their diameter (1.5 cm) and depth (1 cm) differed from those drilled into the rat cheeseboard so that the mice could comfortably consume rewards from them. In the present work, we used chocolate milk as reward, which was obtained by dissolving 10 g of cocoa powder (Clever Trink Kakao, article number 00-425400) in 25 ml of condensed milk containing 7.5% of fats (Clever Kondensmilch, article number 00-426612). In all of the recording, pretraining and habituation sessions, diluted reward (1:5 in distilled water) was dispersed over the whole cheeseboard evenly to eliminate odor-based navigation and to make the surface attractive.

To start the habituation and the pretraining, the mouse was placed on the board for trials of 10 min until no overt sign of nervousness was observed. In the next step the animal was placed in the start box, 100 µl of reward was placed in three proximal wells of the board, these wells were indicated by a visual cue and the door of the start box was opened to let the animal complete a maximally 10-min-long pretraining trial. In all cases, the visual cue was a Falcon tube turned upside down, placed right next to the well with the reward. Our observation showed that within the applied illumination conditions used, the eyesight permitted the C57/BL6 mice to perceive the cue from a distance of ∼15 cm corresponding to three holes on the cheeseboard. To motivate the animal to home, the experimenter placed additional 50-µl reward into the start-box after the mouse left it to search around on the board. When all three rewards were consumed in one trial, the amount in the wells was decreased to 50 µl, and the positions of the rewards were gradually shifted to central and distal locations (i.e., further away from the start-box door). Finally, some trials were conducted without the visual cue. This kind of pretraining typically took one week and 30 trials. The pretraining was stopped when all three rewards were consumed consistently within 10 min even if all placed to distal locations.

One week after the implantation, while the tetrodes were being lowered toward the stratum pyramidale, the animal underwent a couple of reminding trials with all five cables plugged in, and the counterweight of the pulley system (Fig. 6*A*) was adjusted to its body weight. At this stage, the pretraining was stopped for the day after four trials if the animal consistently consumed all three rewards randomly placed on the board. We thoroughly inspected whether the mouse displayed the same freedom of movement at the edge of the board as in the center (Fig. 6*B*,*C*) during this pretraining period, and if necessary, the counterweight was readjusted. In the last evening before the recordings the position of the tetrodes was fine-tuned into the center of the stratum pyramidale; furthermore, the field responses to the laser were checked and the power of the laser was set to trigger maximal field (LFP) responses achievable without saturating the electrophysiological signal. Correspondingly, the laser power was set at a value corresponding to 15 mW of light power at each of the fiber tips (typically 80% of the maximum laser power).

Recording days were either assigned to the sSWR-blockade condition with the light being delivered at sSWR onset instantaneously or to the control condition with the light being delivered 1.32 s after the ripple detector signal. The recording started up with 20 min of sleep/rest in the homecage placed over the board on a stand, followed by 10 min of exposure (“preprobe”) on the clean board sprayed with diluted reward but without baited wells and a block of four learning trials with three baited wells with the visual cue (“1st block”). Before each trial, the board was rotated by 90°, cleaned with ethanol, dried, and sprayed with the diluted reward. The reward positions were kept unvaried but differed sufficiently from positions used on other days. This block was followed by 3 h of sleep/rest in the homecage with optogenetic intervention. Thereafter further four identical learning trials without the visual cue (“2nd block”) and one further identical 3-h-long sleep/rest session succeeded. The memory of the animal was then assessed during a 10-min-long postprobe in the absence of rewards. The postsleep lasted 20 min and was exempt from any optogenetic intervention to avoid the light effects on unit activity so that spikes of the neurons in the dataset can be isolated unequivocally and the recorded population can be analyzed later. The final 30-min-long sleep/rest session served as a technical control with regular half intensity laser pulses to assess the efficiency of inhibition at single cell level.

All three animals did once the SWR-blockade paradigm and once the control paradigm, in varying order. Behavioral data are presented below from a total of six recording days.

## Results

### Expression system for the optogenetic actuator

Behavioral training, electrode adjustment, and recording took several weeks in our experiments. Therefore, it was ideal to have constant levels of the optogenetic actuator protein without having to wait for the ramping up of the AAV-mediated viral expression that can typically take weeks (Aschauer et al., 2013). Furthermore, to inhibit a larger brain region completely, such as the CA1, with the aim of studying the behavioral effects, AAV-based methods can prove to be less appropriate given the uneven and, in some cases, limited diffusion of the viral particles.

**Figure 1.**
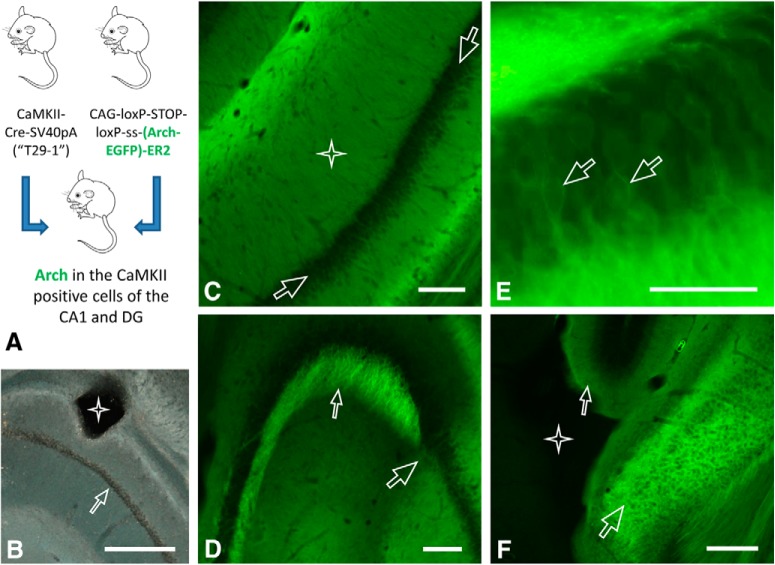
Activation and expression of the transgene. Targeting of the optic fibers. ***A***, The mice expressing archaerhodopsin in the hippocampal CA1 cells were obtained by crossing the homozygous transgenic Tg29 (T29-1) strain expressing the Cre recombinase in the targeted cells with the homozygous Ai35 strain carrying an archaerhodopsin-EGFP transgene at the *Rosa26* locus that can be activated by the Cre recombinase ***B***, Dark-field image of a paraformaldehyde-fixed, unstained section showing the targeting of the optic fiber into the stratum oriens. The trace left by the fiber (four-point star) is visible above the stratum pyramidale (arrowhead). ***C***, The CA1 area of the hippocampus, the four-point star indicates the startum radiatum and the startum pyramidale is visible between the two arrows. Of note, the perisomatic region of the pyramidal cells contains little of the exogenous protein while their dendrites light up, consistently with the membrane targeted expression. The strong green signal in the CA1 is derived from the apical and the basal dendrites of the pyramidal neurons in the stratum pyramidale. ***D***, Comparison of the expression in CA1 and CA3. The large arrow points to the CA2 on the right hand side of which the CA1 shows strong expression. On the left hand side the cells of the CA3 show minimal expression with the stratum lucidum lighting up (small arrowhead) as a result of strong expression in the granule cells of the dentate gyrus. ***E***, A close-up on the lower blade of the dentate gyrus, where membrane localization can be suspected even when using traditional light microscopy (similar membrane localization was seen in the CA1 region when the Arch-EGFP transgene was activated only in a few CA1 cells in a different genetic intercross) The two arrowheads point to two cells with a suspected stretch of Arch-EGFP rich plasma membrane. ***F***, The CA1 fibers terminating in the subiculum (large arrowhead) and the stratum moleculare of the dentate gyrus (small arrowhead) express strongly the gene construct, and this can be compared to the extremely low level of expression in the subcortical structures (four-point star). Scale bars: 100 µm (***C***, ***D***) 200 µm (***E***, ***F***), and 500 µm (***B***). ***C–F***, EGFP fluorescence is visualized using light microscopy

Given these constraints, we have opted for a stably integrated transgene coding for the optogenetic actuator instead of viral injections. Therefore, all the mice included in this study were obtained from breeding pairs consisting of one homozygous Ai35 (Madisen et al., 2012) and one homozygous Tg29-1 (Tsien et al., 1996) animal (Fig. 1*A*). The Tg29-1 mouse line has already been used in several hundreds of studies and carries a CaMKII-Cre transgene that directs the expression of the Cre recombinase to forebrain tissue (Tsien et al., 1996). Notably, in the hippocampus proper, these animals express Cre in the CA1 region and the dentate gyrus (Pieraut et al., 2014) but show minimal, if any, Cre activity in the CA3 region.

The Ai35 line has been generated more recently and carries an archaerhodopsin allele (CAG-LoxP-STOP-LoxP-archaerhodopsin-EGFP) at the *Rosa26* locus (Madisen et al., 2012) that is nearly completely exempt from epigenetic effects spreading in from neighboring genomic sequences.

In forebrains of mice obtained from Tg29-1 × Ai35 crosses the steady-state levels of the archaerhodopsin-EGFP were reached early, before one month of age. Accordingly, the expression levels were stable and were uniform in the targeted cell populations and also among animals. As a further advantage over viral transduction, which can trigger cell death not only by intrinsic effects but also by resulting in unwantedly high protein expression (Howard et al., 2008), we did not observe any toxicity and always saw modest levels of the exogenous protein (Fig. 1). Therefore, we used the progeny exclusively from homozygous Tg29-1 and Ai35 parents in the present work.

### Designing the optogenetic microdrive

**Figure 2.**
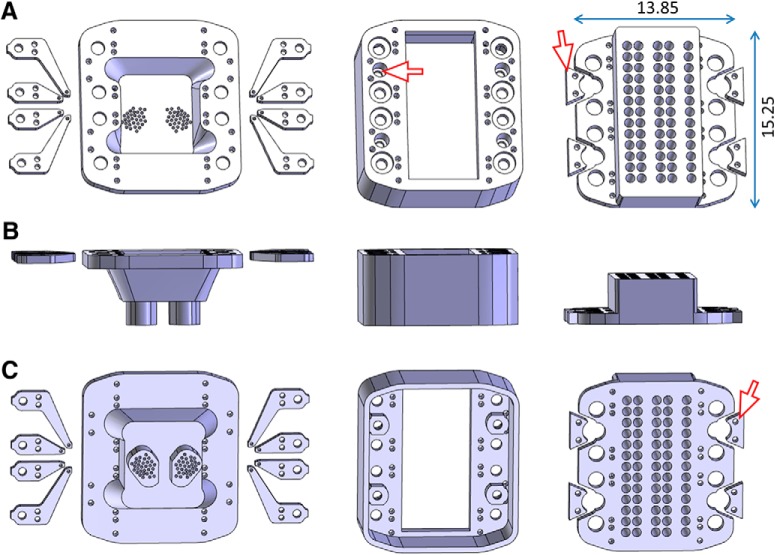
Designing the microdrive. ***A***, Top view of the pieces, from left to right: left shuttles, bottom part, right shuttles, middle part, and lid. The red arrowhead points to the annulus (inside the socket for the ferrule) that avoids the ferrule being pressed into body of the microdrive. Main dimensions are indicated in millimeters. ***B***, Occipital view of the same pieces as in ***A***, in the same order. ***C***, Bottom view of the same pieces as in ***A***, in the same order. The arrowhead points to the locking piece that slides into the diced groove of the ferrule and is locked in position by two insect pins to avoid the ferrule being torn out when the mating sleeves are pulled off to disconnect the animal from the laser.

Taking together all the requirements outlined in the previous sections, we started from a closed, captive screw microdrive design (Ainsworth and O’Keefe, 1977). Our concept was based on three main microdrive pieces (bottom part, body, and lid) held together by insect pins and harboring eight shuttles that move steel cannulae with tetrodes independently up and down (Fig. 2*A*, left and right shuttles are shown on the left and right side of the bottom part of the microdrive, respectively). Our aim was to incorporate four optic ferrules in a stable manner so that the frequent plugging and unplugging of the optic cables before and after the recording sessions would not deteriorate the efficiency of the light transmission.

**Figure 3.**
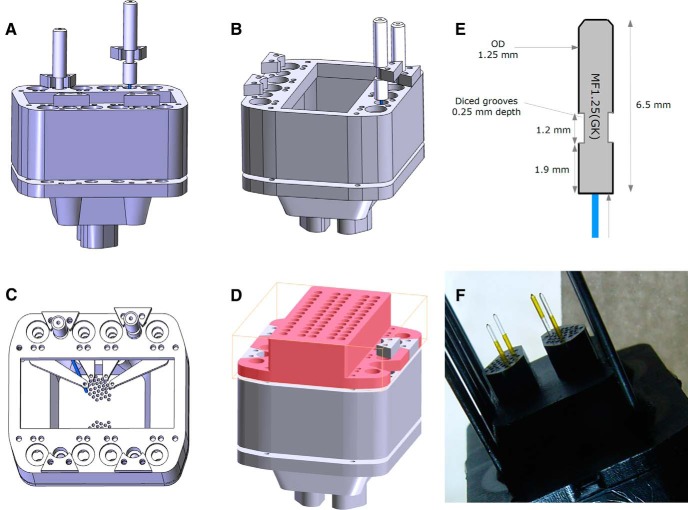
Integrating the optic system into the microdrive. ***A***, The assembled bottom and middle parts without insect pins, viewed from right side of the animal. Two locking pieces on the left side of the animal are slid into the grooves of the ferrules, the other two are simply indicated in their final position. ***B***, Same assembly as in ***A***, viewed from the front. ***C***, Same assembly as in ***A***, viewed from the top. Only the left occipital optic fiber is shown in full length, guided between the shuttles into the desired electrode hole. ***D***, Fully assembled microdrive (for sake of clarity, insect pins are not shown, and the lid appears in pink). ***E***, Dimensions of the metallic optic ferrule (gray) with an integrated PMMA optic fiber (blue). ***F***, Photo of a microdrive with four optic fibers protruding from the electrode holes, three of them having polyimide tubing (yellow) in final position, while the tubing on the forth fiber is being mounted. Insect pins are visible on both sides. Note the conical tip of the PMMA fibers.

Therefore, on each side of the microdrive body, we placed two 1.9-mm-deep sockets for the ferrules, with an annulus at the bottom (Fig. 2*A*, red arrowhead). This way the ferrules could firmly sit onto the annulus and resist the pressure of plugging the optic cables before the experiments while the optic fibers inside the drive could still pass through the annulus. A 1.2 mm high and 0.25 mm deep diced groove was etched into each optic ferrule (Fig. 3*E*) and after closing the microdrive with the lid, one supplementary piece (Fig. 2*A*,*C*, red arrowhead) was slid into the groove of each optic fiber and locked in position with two extra insect pins spanning the whole body of the microdrive. This solution prevented the ferrule from being torn out from the microdrive when the optic cables were disconnected after the experiments.

**Figure 4.**
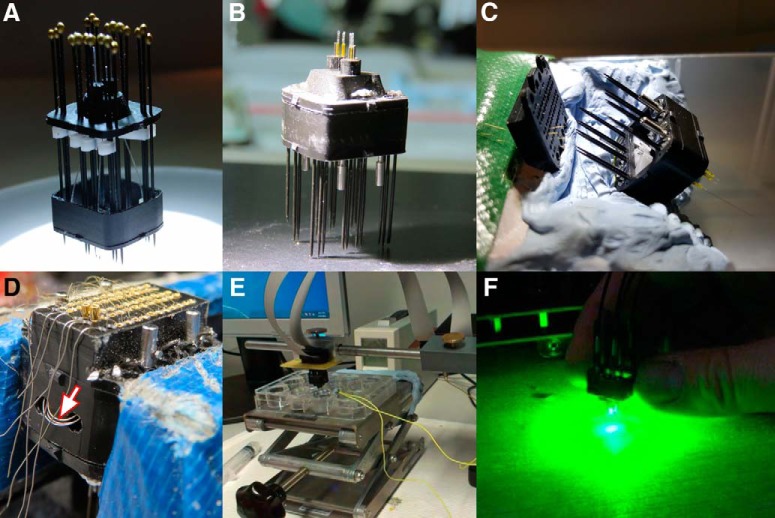
Building the microdrive. ***A***, Bottom part (black), shuttles (white), middle part (black) held together by the insect pins. The optic system is fully integrated at this stage, but the ferrules are not visible since the assembly is viewed from the bottom. The shuttle screws are not yet in position (that would hold back the shuttles inside the middle part). ***B***, The steel cannulae are already inserted, the microdrive is fully closed, the blunted ends of the insect pins are bent and cut away and the polyimide tubing is mounted onto the optic fibers. ***C***, The microdrive is being loaded with tetrodes. Four individual microwires are guided through the desired holes of the lid, the corresponding tetrode is visible on the right hand side, coming out of its cannula. ***D***, The drive is placed in a vice (blue), the sharp end of the insect pins is bent and then cut away. The contactor pins are being pushed into position, the tetrode wires and the thicker ground wires are visible as coming out through their holes in the lid. A loop formed by the same ground wires, guided out through two holes in the middle part is shown by a red arrowhead. Ground wires from the occipital skull screws will be soldered to this loop during implantation and the loop will be buried in cement. ***E***, The cut surface of tetrodes is being gold plated by directing current through each channel individually while the tetrodes are dipped in Au(III) solution. ***F***, The light transmission of the drive (at stage ***B*** of this figure) is verified by plugging it directly to the laser source via the four daughter cables that will be used for recording as well. This procedure can also be completed with a fully finished drive (at stage ***E*** of this figure) and the amount of light transmitted by each fiber can also be measured individually.

To guide the light from the ferrules to the brain inside the drive, we opted for 16-mm-long PMMA optic fibers (Ø = 250 µm; Figs. 3*E*, 4*A*) that have several advantages over silica fibers: (1) stronger bending is possible inside the body of the drive, (2) the numerical aperture value is higher (NA = 0,65) permitting the transmission of more light into the brain, and finally (3) the etching of the tip into a cone (α = 45°) is more convenient. At implantation, the conical tips displaced the small mouse brain much less than cylindrical tips, and, additionally, distributed much better the light over the whole dorsal CA1 pyramidal cells when placed into the stratum oriens, 150 µm above the stratum pyramidale (Fig. 1*B*). The stiffness of the approximately 3-mm-long stretch of the fiber protruding from the bottom of the microdrive was suboptimal to penetrate uprightly into the brain during surgery; therefore, a 1.5-mm-long portion was covered with silica tubing (Fig. 3*F*) glued to the bottom part of the microdrive and also to the fiber surface. This way the counterforce exerted by the brain tissue during implantation did not push the fibers back up into the body of the microdrive.

Although the PMMA fibers are much less rigid than silica fibers, it was still the case that when the sockets for the optic ferrules were placed into the corners of the microdrive, excessive bending damaged the optic fibers and frequently led to rupture. Thus, we reallocated the positions for the screws (S) lowering the shuttles and the sockets for the ferrules (F) and chose an SFSSFS layout, which implied that the shuttles in the corners were also redesigned to have an unusually long shaft (Fig. 2*A*,*C*). This way, the estimated curvature of the optic fibers inside the drive was between 25° and 35° and depended on the position (rostral or caudal).

Finally, in some initial experiments, we observed that with fully and correctly connected optic cables the laser light leaked, partially via the body of the optic ferrule, and was clearly visible to the mice. To circumvent this leakage, we substituted metallic optic ferrules for the ceramic ones and covered the female interconnectors (so-called “mating sleeves”) linking the optic cable and the ferrule with a thin sheet of black rubber.

### Building the optogenetic microdrive

Assembly of our optogenetic mouse microdrive is typically very time-consuming for experimenters without experience, and some typical errors inevitably result in having to discard the whole device (including all the optic ferrules and optic fibers) which leads to losing a lot of time and resources. Therefore, in the following section, we describe the detailed building procedure that can also be used as a protocol transferable to any laboratory and is typically completed by an experienced user in 2 d, with most of the steps done under a binocular microscope.

1) To hold the three parts of the drive together, insect pins (Fig. 4*A*) are used which, at the same time, serve as tracks for the shuttles to move up and down. The first step of the assembly is to insert these pins into the bottom part of the microdrive. There is a total of 20 pins to insert and the head of each pin should point towards the bottom of the drive (Fig. 4*A*). They should come out of the bottom part by about 1.5 cm. There are eight additional pins that lock the optic ferrule in its position, these are to be inserted at a later step.
2) Each of the shuttles is mounted on two insect pins (the pins located in the corner are not used for this purpose); these will allow the experimenter to move the electrodes independently up and down. The shuttles come in two different shapes: short and long (Fig. 2*A*,*C*, and appear in white in Fig. 4*A*). The short ones go in the middle of the drive and the long ones at the extremities. Care needs to be taken not to destroy the smallest hole at the tip of the shuttle which is intended to hold its steel cannula.
3) The middle part of the microdrive can optionally be pierced for the ground wires on its occipital side (holes visible in Fig. 4*D*) and has to be pulled onto the insect pins (Fig. 4*A*). The optic ferrules with integrated fibers have to be placed in the sockets of the middle part while gently guiding the optic fibers into the desired hole of the bottom part with padded forceps.
4) The drive is closed completely and the shuttles are lifted up by their screws (four are to be used on each side, there is one for each shuttle; their heads are visible in Fig. 4*C*).
5) Each steel cannula is pulled through the hole located at the tip of its own shuttle and is guided through the desired hole of the bottom part using appropriate forceps. For performing this action, it is essential that the drive be inverted as compared to its position in Figure 4*A*, and a light source be placed under the drive so that all the holes can clearly be seen. Furthermore, the drive needs to be reopened at this stage by slowly inserting a screwdriver between the bottom part and the middle part so that the cannulae can be accessed with forceps, an approximately 2-mm-wide opening is required. Normally, the cannulae need to be bent slightly to target the desired hole and this is sufficient to hold them temporarily in position.
6) The cannulae are carefully glued to the shuttles, and the optic ferrules to the sockets, using a small amount of Superglue gel. After solidification, the drive can be closed completely by bending the head of the insect pins and cutting their head away with a miniature cutting disc (Fig. 4*B*). The contact between the insect pins and the bottom part is reinforced by applying a thin layer of Superglue gel on the bent stub of the insect pins.
7) Using padded forceps, the optic fibers can now be adjusted to have their tips at the same position, by gently pulling rather than by pushing. When in position, silica tubing can be pulled onto them to cover the exposed stretch that is not going to penetrate into the brain. Liquid Superglue is used to fix the tubing to the bottom part and is also guided between the tubing and the optic fiber.
8) To prepare the next step, 14 tetrodes (to be used in pairs in their cannulae) are prepared using 12-µm insulated fine wire, and one single tetrode using 17-µm fine wire using standard tetrode-fabrication techniques (Gray et al., 1995). The drive is loaded by laying the lid upside down very close to one row of the insect pins sticking out from the middle part of the drive (Fig. 4*C*). Subsequently, the tetrodes are guided through the cannulae, either as single tetrodes (17 µm) or as pairs (12 µm). The individual wires are then sorted out into the appropriate holes of the drive lid, and each tetrode or the pair is glued into its cannula using low-viscosity liquid Superglue that flows inside by capillarity. The liquid Superglue has to let flow around the cannula as well; this way it also reinforces the contact between the cannula and the shuttle. Finally, the drive is closed by pulling the lid onto the insect pins and sliding it down gently and slowly. Before complete closure, the ground wires have to be guided through the holes of the lid, and through the 2 holes drilled into the middle part to form an accessible loop (Fig. 4*D*, red arrowhead).
9) To lock all four optic ferrules in position, the supplementary locking pieces (Fig. 2*C*, red arrowhead) are slid into the diced grooves, and held there in position, while for each ferrule two extra insect pins are poked through the whole body of the microdrive. A small amount of liquid Superglue is guided so as to flow down along the ferrule without smudging the connector surface.
10) The drive is placed in a vice so that the contactor pins can be pushed into the lid and the overhanging portion of the insect pins can be bent and cut away (Fig. 4*D*). It is of utmost importance to drive down the shuttles to the lowermost position for this step so that the wires do not break later at repositioning. The insulation is removed from the tetrode wire in this step by the gold pin being pushed in since it squeezes the wire against the side wall of the hole in the lid. Again, the bent stubs of the insect pins are covered by a thin layer of Superglue gel without blocking the shuttle screw holes and the diced grooves of the optic ferrules. Finally, a small drop of soldering material is placed onto the ground wire loops and the contact of this drop with the ground pins (five occipital gold pins in the drive lid) is verified.
11) Light transmission through the drive is checked by plugging it to the laser. The performance of the four fibers can be assessed independently (Fig. 4*F*). At each of the optic fiber tips, the light power should be minimum 15 mW at 80% laser power (561 nm). For sSWR blockade experiments like the in the present report, the optimal power output does not exceed 15 mW at each of the optic fiber tips. Therefore, if the light power is higher, the laser has to be adjusted accordingly.
12) The drive is turned upside down again under the binocular microscope. A small amount of low-viscosity Superglue is applied into the cannulae to glue the tetrodes into them, this will markedly increase recording stability. Subsequently, the tetrodes are cut to the desired length, and those in pairs are gently divided with precision forceps without introducing bending. The separation is locked at an angle of approximately 15° with Superglue close to the cannulae, this is necessary to prevent recording of the same neurons on both tetrodes of the same pair.
13) On the day before the surgery, the drive is gold plated by plugging it into the appropriate adaptor and dipping the tetrode tips into Au(III)-containing solution to deposit gold electrolytically, thereby reducing the channel impedances to 250–300 kΩ. Ideally, plating-impedance measurement cycles are carried out for each channel with a software such as NanoZ (Fig. 4*E*). Subsequently, the tetrode tips are washed in saline solution and the impedances are remeasured.
14) The shuttles are driven up to the highest position, or to a position that is high enough to target the desired structure in the brain. Specifically, in the work presented here, the tetrode tips were placed 150 μm above the conical tip of the optic fibers.

### Implanting the optogenetic microdrive

**Figure 5.**
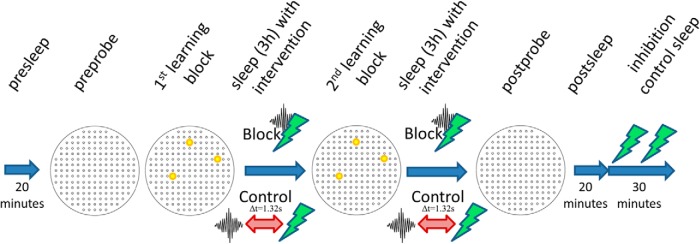
The structure of a recording day. The preprobe was 10 min long and with the mouse moving around on the cheeseboard without rewards being presented. There were four learning trials in both (1st and 2nd) learning blocks and three rewards were presented in all of them, always in the same three positions within a recording day. During the 1st block, signposts placed right next to the baited holes also guided the animals. The optogenetic intervention (80% laser power) during the 3-h-long sleeps was either SWR-blockade (upper row) or control intervention (lower row). The postprobe was 10 min long, testing the memory retention of the mice, therefore no rewards were presented. The postsleep was used to assess the reactivation of the CA1 pyramidal neurons. The final 30-min-long sleep with regular laser pulses (at 50% laser power) served as a technical control to measure the efficiency of the optogenetic inhibition at single cell level.

**Figure 6.**
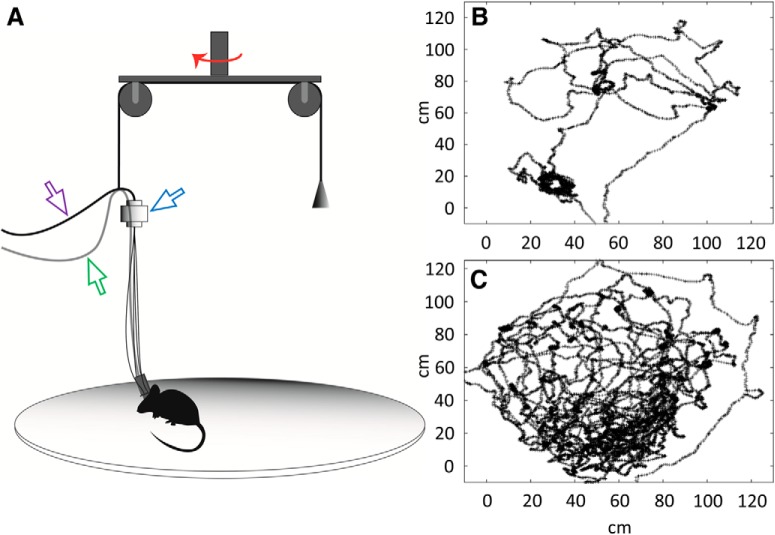
Elements and efficiency of the pulley system. ***A***, Elements of the pulley system: a counterweight was used to balance the weight of the headstage preamplifier (visible at the head of the mouse), the rotatory joint transmitting the laser light (blue arrowhead), the optic cables (purple arrowhead), and the recording cable (green arrowhead). The two pulleys could turn around a common axis in the horizontal plane (red arrow). ***B***, Tracking data from a learning trial, showing the position of the animal until the moment of reentering the start-box that is located at the bottom of the figure (the area of the start-box is not included). ***C***, Tracking data from a postprobe (duration: 10 min) showing that the movement of the animal nearly completely covers the cheeseboard.

Animals pretrained on the cheeseboard were implanted according to standard procedures, similar to those used for other drive types and for simple optogenetic or head-fixed experiments (O’Neill et al., 2006; the details are provided in Materials and Methods). The layout of the optic fibers and tetrodes corresponded exactly to the one used in our previous report (Kovács et al., 2016, their Fig. S4). After a week of recovery, the animals underwent reminding trials that prepared them for the experimental paradigm (Fig. 5), while the tetrodes were being lowered toward the stratum pyramidale. During the reminding trials, the counterweight of the pulley system (Fig. 6*A*) was readjusted to ensure that the animal has the optimal freedom of movement (Fig. 6*B*,*C*) and it does not sustain either a pulling force at the edges of the board or a pushing one at the center.

### Recordings from behaving mice coupled with optogenetic inhibition of sSWRs

One main goal that drove us to develop the optogenetic microdrive described here was to better understand the role of sSWRs in learning. A straightforward approach to delineating the role of such events is to fully block them and then verify whether any aspect of learning is different on such blockade. We detected sSWRs in sleep/quiet immobility sessions using an analog bandpass filter device, which triggered our laser device that in turn inhibited CA1 neurons via constitutively expressed exogenous archaerhodopsin channels. We aimed to illuminate the entire dorsal CA1 area bilaterally using four optic fibers with optimized conical tips.

In a previous work, we used this technique (Kovács et al., 2016) to investigate the effect of sSWRs-blockade on the stability of novel CA1 place maps but, using a passive open field exploration paradigm, we did not observe differences between the sSWRs blockade and control conditions (but see van de Ven et al., 2016). We attributed these negative results to the passive nature of the open field exploration and to confirm this assumption, we have now chosen a goal learning task on the cheeseboard maze (Dupret et al., 2010), because it relies on active reward-seeking behavior and spatial reference memory. Given that this task requires the free movement of the animal over a large area, it was also suitable to test the applicability of our optogenetic recording system and microdrive to complex behavioral paradigms.

In the cheeseboard task, rewards were hidden during learning trials in three out of 177 holes of a rotatable disk with a diameter of 120 cm (Dupret et al., 2010). In the course of the probe trials, no rewards were presented and the persistence of the mouse to seek at positions where the rewards were previously located was quantified. During all trials, we have counterbalanced the weight of the headstage preamplifier (on the head of the animal) and that of the rotatory joint (hung above the animal, used to distribute the laser light into four daughter cables) with the pulley system (Fig. 6*A*). Notwithstanding the force the mice had to exert to pull all the five cables, we did not observe any location-specific bias in the speed of moving or in the amount of stalling over the whole cheeseboard, which clearly demonstrates the potential of our microdrive and our recording system to be used in any kind of complex mouse behavioral task.

**Figure 7.**
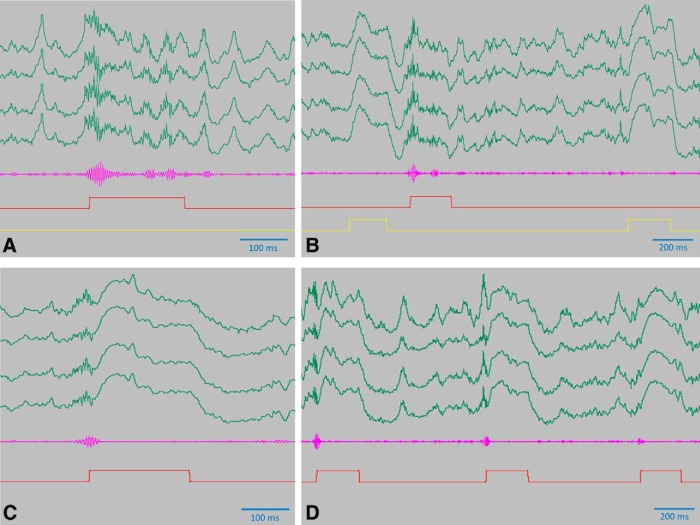
Efficiency of the SWR-blockade. ***A***, ***B***, Detector signal and delayed light pulses in the control condition. Raw signal from one tetrode (green), bandpass filtered differential signal (for the details of signal processing, see Kovács et al., 2016) fed into the detector (magenta), the 200-ms-long detector signal (red), and delayed signal driving the laser (yellow) is shown. The first delayed signal visible on the left hand side belongs to a previous detector pulse not appearing in the figure. During the 1st 20 min of the 1st 3-h-long middle sleep of a control day, 319 SWR events were detected online (established by counting the detector pulses), out of which nine (2.8%) were undetected by an offline algorithm that found 14 additional SWR events (4.4%). ***C***, ***D***, Detector signal in the SWR blockade condition. Color coding is the same as for panel A and B. The signal coming from the ripple detector is directly driving the laser here, therefore no delayed signal is used. During the 1st 20 min of the 1st 3-h-long middle sleep of a control day, 332 SWR events were detected online (established by counting the detector pulses) out of which 298 (89.7%) were undetected by the offline algorithm, a high percentage to be attributed to the fact that these events themselves were nearly fully destroyed by the light pulses. The offline algorithm detected 11 additional events (3.3%) that were undetected by online algorithm during the intervention. ***A***–***D***, Note the field responses that are generated by the light pulses and are visible as strong positive deflection of the LFP. Also note the destruction of the oscillation close to the onset of the detector pulse in ***C***, ***D***, and the persisting oscillation in ***A***, ***B***.

To verify the efficiency of the SWR-blockade we have recorded the first 20 min of the two 3-h-long sleeps, that followed the 1st and 2nd block of learning trials, respectively. These recordings showed that the instantaneous delivery of the laser light at the SWR onset readily destroyed the oscillation (Fig. 7*C*,*D*), while the delayed delivery of the light used in the control condition left enough time for the SWR events to develop (Fig. 7*A*,*B*). Strong field responses could always be observed coincidently with laser light delivery (Fig. 7), consistently with archaerhodopsin activation in a large population of neurons and the concomitant increase of the LFP.

**Figure 8.**
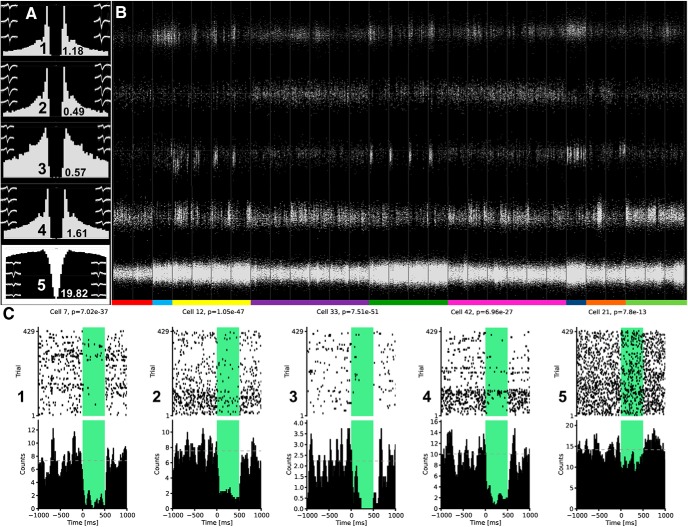
Stability and optogenetic inhibition at single neuron level. ***A***, Interspike interval distributions (temporal autocorrelograms) and firing rates of five selected cells: putative pyramidal cells from the septal pole of the right CA1 (1), the septal pole of the left CA1 (2), the temporal pole of the right CA1 (3), the temporal pole of the left CA1 (4), and a putative interneuron from the CA1 (5). The first and the last hundred waveforms measured during the whole period in B are overlaid and shown as left and right insets, respectively, for each of the five neurons. ***B***, Isolated spikes from the same five cells as in panel A during the entire recording day. The structure of the behavioral paradigm has a clear effect on the firing of the cells. To illustrate this effect, the bar at the bottom of the panel identifies the elements of the paradigm: presleep (red), preprobe (light blue), 1st block of learning trials (yellow), 1st sleep with intervention (purple), 2nd block of learning trials (light green), 2nd sleep with intervention (magenta), postprobe (dark blue), postsleep (orange), final sleep for the assessment of the inhibition (light green). ***C***, Optogenetic inhibition of the five presented cells (same ones as in panels ***A***, ***B***) during the final 30-min-long sleep shown in panel ***B***. During this sleep, regular, 500-ms-long laser pulses were delivered every 4th second. In the upper half of the panel, spike rasters cover the 28.6-min-long middle period of the sleep, in the lower half of the panel, these rasters are summed up to create a histogram; *p* values derived from the Wilcoxon signed rank sum test is indicated above each raster.

To demonstrate that our microdrive is suitable to record sufficiently stable units even in long and complex behavioral paradigms, we present two cells from septal pole (B = -1.75) of the right and left CA1 (Fig. 8*A–C*; first and second cell) and two cells from the temporal pole (B = -2.75) of the right and left CA1 (Fig. 8*A–C*; third and fourth cell). The efficiency of the inhibition was tested at single cell level at the end of each recording day by delivering regular 500-ms-long pulses, at each 4th second, during a 30-min-long final sleep/rest session. Efficiency was assessed by comparing the number of spikes during the totality of the 500-ms-long inhibition periods to the number of spikes during the totality of the 3500-ms-long inhibition-free periods (Fig. 8*C*), the same way as in our previous publication (Kovács et al., 2016). According to this assessment only the interneurons, which did not express the optogenetic actuator, were left uninhibited (Fig. 8*C*) and slight changes of their activity could only be attributed to network effects while pyramidal cells were substantially inhibited (Fig. 8*C*) regardless of their anatomic position.

**Figure 9.**
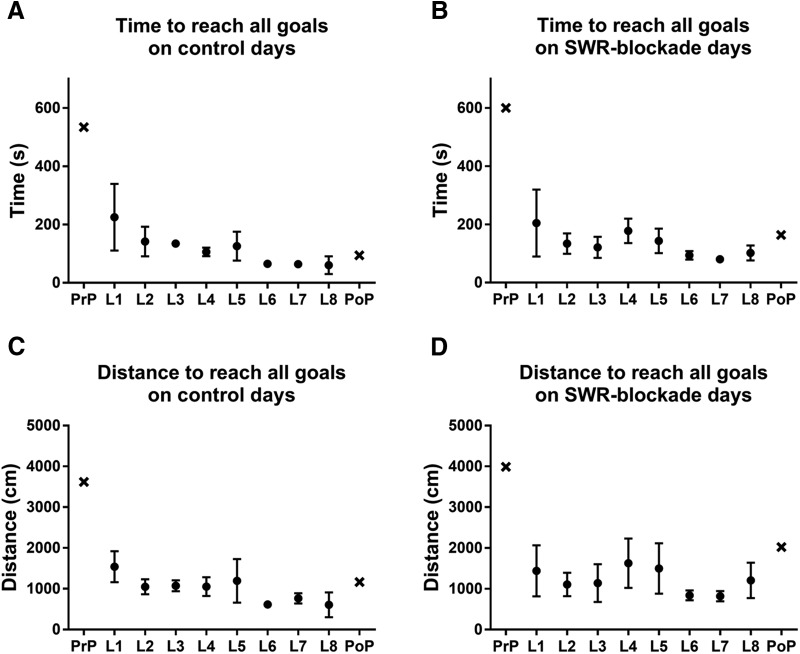
Behavioral performance in the cheeseboard task. ***A***, ***B***, Overall time spent on control days (***A***) and on SWR-blockade days (***B***) until reaching all the three goal locations. Mean values are shown for three recording days. ***C***, ***D***, Overall mean distance traveled on control days (***C***) and on SWR-blockade days (***D***) to reach all the three goal locations. ***A*–*D***, The preprobe (PrP) and the postprobe (PoP) are both indicated (cross), L1–L8 stand for the eight learning trials (filled circles) with rewards provided. Error bars representing the SEM are included to give an insight into the variability but are derived from only three data points.

Our paradigm consisted of two learning blocks of four learning trials on the cheeseboard with chocolate reward and a probe trial of 10 min without any baited location (Fig. 5). Of note, our paradigm differed from Dupret et al. (2010) in that in the first four trials, but not in the remaining ones, local cues guided the animal to find food. Two sleep/rest sessions, each one lasting for 3 h, one between the two learning blocks and the other between the last learning block and the postprobe session, were used to block sSWRs with a 200-ms-long laser light pulse (Fig. 5). On control recording days, the same paradigm was used, with the sole difference that mock blockade was delivered using delayed pulses that lagged by 1.32 s behind the sSWRs that triggered them (Fig. 5). In the course of these experiments, the animals learned the locations of the three chocolate rewards as evidenced by the learning curves, plotted as the time taken (Fig. 9*A*,*B*) and distance traveled (Fig. 9*C*,*D*) to reach all three goal locations. To quantify the effect of the sSWRs blockade, the dwell time and the number of crossings at the three goal locations were computed during the postprobes for the true sSWRs blockade (three animals) and mock blockade (same three animals). The behavioral performance showed a strong tendency of decline when the sSWRs were eliminated: the mean ± SEM of the corssings counted were 6.696 ± 2.047 and 1.820 ± 0.587 for the control and SWR-block condition, respectively, while the same for the dwell time was 7.111 ± 1.033 and 3.333 ± 0.9574. Given the low number observations (nine for each condition) the postprobe sessions yielded, the observed level of difference is remarkable and suggests that the elimination of sSWRs interferes with the retention of the recently learned goal locations and sSWRs are needed for the stabilization of spatial memory traces of fixed goal locations. Nonetheless, in the future, a larger number of mice will be needed to formally assign this role to the sSWRs which was beyond the scope of the present study focusing on the technical aspects.

To investigate the hippocampal plasticity processes that might underlie our behavioral findings, we also collected single unit data from the tetrodes located in the dorsal CA1 area (Fig. 8). We isolated those putative principal neurons from the three animals that showed activity all along the behavioral paradigm, this way we could identify 60 and 76 pyramidal cells in the sSWR blockade and in the control condition, respectively. Analyses based on these cells will be published in a separate report.

## Discussion

In the present work, we developed new methodology for the optogenetic manipulation of a large volume of brain tissue *in vivo,* performed jointly with massively parallel recording from freely behaving mice. We used the new tool to get an initial insight into whether sSWRs contribute to spatial learning in a complex reward-guided goal learning memory task.

### The efficiency of the novel mouse optogenetic microdrive

Recording coupled with optogenetics in the mouse constitutes a significant challenge since the weight of a C57/BL6 mouse typically varies around 25 g. Correspondingly, the size and the weight of the cables and the implant continues to be a critical limiting factor in these experiments, along with the restraining effects on the exploratory head movements and even on the straight-ahead running of the mice.

Our 60-channel optogenetic microdrive, when implanted with a moderate amount of cement, can burden the head of the mouse with as little as 3-g extra weight, whereas published 16-channel mouse microdrive systems even without incorporated optogenetics can weigh as much as 1.5–2.0 g (Chang et al., 2013). Moreover, the size of the implant is convenient and does not protrude either to the front or to the side of the skull, thereby letting the animal explore edges and corners of mazes even when higher walls surround them. The task we used to test the recording system involves exploring over a circular board having a diameter of 120 cm; nonetheless, even when the trajectory covered the board completely, we did not observe the animals having any difficulty to move continuously (Fig. 6*B*,*C*). Furthermore, even at food wells located at the very edge of the board, the animals could lower their head easily to take out rewards from the wells. These observations indicate that neither the weight of the implant nor any force exerted by the five cables plugged into the microdrive (Fig. 6*A*) unduly restricted the movement of the mice.

In shorter recordings, our microdrive yielded a large number of well isolated simultaneously recorded CA1 units. However, the number of units active all over the cheeseboard paradigm was substantially lower for two main reasons: (1) the sophisticated behavioral pretraining of the animals could not be harmonized in every case with the lowering of the tetrodes and screening for CA1 units, (2) the extreme length (close to 9 h) of the recording days resulted in some neurons appearing or disappearing completely.

The extent of the optogenetic inhibition was estimated in Figure 8*C* using the method presented in a previous study from our laboratory (Schoenenberger et al., 2016). At the single cell level, we observed strong, near complete inhibition that did not depend on the anatomic position of the pyramidal cells (Fig. 8*A–C*). At the population level, the inhibition was evidenced by strong field responses that readily destroyed ripple oscillation (Fig. 7). Therefore, we concluded that four optic fibers, spreading the light laterally from their conical tips, were sufficient to bring about inhibition over a large brain area such as the dorsal CA1 region that is close in dimensions to 2 × 2 mm.

### Initial observations on spatial memory formation in a complex paradigm

The two-stage model of memory formation suggests a role for sSWRs in memory consolidation (Buzsáki, 1989). The particular element of this concept, which emphasizes that during slow-wave sleep subsequent to exploratory activity sSWRs transmit information to neocortical brain areas, has definitely gained further experimental support since its original proposal (Logothetis et al., 2012; Khodagholy et al., 2017).

Correspondingly, many experimenters decided to block SWR-related physiologic activity either during slow wave sleep or during awake periods. When sSWRs were disrupted by electric stimulation during post-training consolidation periods, the performance of rodents decreased in the eight-arm radial maze (Girardeau et al., 2009) and the wagon-wheel maze (Ego-Stengel and Wilson, 2009) task as shown by the learning curves. SWR can also be observed during awake periods when the animal pauses in the midst of a task and blocking this kind of activity by electric stimulation reduced the spatial memory but not the reference memory of the animal (Jadhav et al., 2012). In a previous study, we observed that blocking sSWRs did not influence the stabilization of a new CA1 population code when mice passively explored a novel environment and were not engaged in active reward-seeking behavior (Kovács et al., 2016). However, a subsequent study observed a deficit in the expression of spatial assemblies in this condition (van de Ven et al., 2016), albeit no control illumination following the sSWRs with a delay was performed there. This raises the possibility that light-mediated inhibition, or other light-related artifacts and not sSWR blockade per se caused the differences in this report.

In the present work, we used a complex spatial task, and we did see strong tendency on spatial learning when we inhibited the whole CA1 at the SWR onset. We think that the difference between our previous study and the data presented here lies in the fact that mice were motivated this time to actively memorize reward locations instead of passively exploring an arena. Nonetheless, to formally prove that the sSWRs are required for post-training memory consolidation in a complex, demanding task, a larger number of mice will have to be tested in a similar behavioral paradigm.

The analysis of single unit data collected here could also contribute to decide whether sSWRs are indispensable for spatial learning. Our findings are in accordance with the electrophysiological results of a team (Roux et al., 2017) that blocked awake SWR events. In that work no attempt was made to disrupt learning performance because their light delivery was optimized to influence only small CA1 microcircuits (Roux et al., 2017).

### Summary

Taken together, we successfully designed and built a compact, light-weight optogenetic microdrive and implanted it into transgenic mice. The device did not restrict the natural behavior of the animals and made us able to inhibit optogenetically a large proportion if not the entirety of the dorsal CA1 at the onset of particular oscillatory events in sleep while successfully recording from a sufficiently large number of neurons of the same brain area throughout a complex 9-h-long behavioral paradigm. Our experiments conducted to test the novel microdrive suggest a role of sSWRs in in promoting spatial learning. We suggest that the methodology presented here will be useful to answer many other questions in the field of experimental/behavioral neuroscience.

## Synthesis

Reviewing Editor: Liset Menendez de la Prida, Instituto Cajal CSIC Decisions are customarily a result of the Reviewing Editor and the peer reviewers coming together and discussing their recommendations until a consensus is reached. When revisions are invited, a fact-based synthesis statement explaining their decision and outlining what is needed to prepare a revision will be listed below. The following reviewer(s) agreed to reveal their identity: Alexey Ponomarenko.

Your ms was evaluated by two reviewers and myself. We had some conflicting views. One reviewer was overall positive, but R1 and myself we feel in its current form your ms requires some additional work. My point of view is that you lack some focus. It is unclear whether this ms is about a technique or about a scientific question (cognitive role of SPW-ripples). In the first case, we feel you could revise the piece and return a revised version in less than two months. Instead, if you want somehow to include data on the cognitive role of SPW-ripples, then you may need much more time. In eNeuro 2 months is a clear cut limit.

After considering these options, we decided to grant you with the possibility to resubmit. However, note that if you still want to include the part of the cognitive role of SPW-ripples much more data and analyses are required and all reviewers and myself have to agree.

I copy below the reports by R1 and R2, as well as may own notes in an independent reading. I hope you find all this useful.

RE notes:

- A major problem I see is that the ms lacks some focus. There are two goals here: a) reporting a new technique (four point irradiation closed loop optogenetics); b) reporting data on the cognitive role of sharp-wave riples obtained with the technique. Somehow it reads unbalances. Authors need to make a decision and revise accordingly. If the main focus is a, revisions would require less than two months. If the message is: thanks to a I have b, then much more data and statistical analyses are required and that would need more than two months (rejection).

- Fig.1 is reflecting an standard procedure, not adding specifically to the method itself. It can be integrated together with content in Fig.2.

- In Fig.2, I wonder about ChR2 expression and specificity. One typical claim of optogenetics is that it is local affecting only the target population. However, the fact that both fibers and dendrites are expressing ChR2 makes stimulation a bit unspecific.

- Raw ephys data are not shown. If the focus is a, then authors need to show how SPW-ripples events are recorded and blocked with closed-loop approaches. They should also somehow validate false +/- events. If the focus is b or a-b, then raw ephys data should be more carefully presented and quantified to support behaviorual results, including measures of co-firing.

- I wonder whether this is an adaptation of a commercially available microdrive (not sure but I think this design is origical from R Muller). So, in order to make the case stronger for a focus in a or in a-b, authors need to reinforce what is novel and different. Also, given that closed-loop is a major part of technique, it should gain more methodological visibility.

- One author is involved in the company producing the device. This should be probably disclosed.

Comments by R1:

The authors present work describing a multi-tetrode recording system for mice that integrates four separate optical fibers for optogenetic manipulation. This device is used to provide closed-loop, widespread optogenetic inhibition to CA1 hippocampal neurons during SWRs and the effects of this intervention are explored in a spatial learning paradigm. I have the follow concerns:

MAJOR

- The manuscript is as is too skeletal. Important additional information should be included, including the following:

- Methodological details, including what antibiotics, anti-inflammatory, and auxiliary analgesic compounds were given; how long surgeries lasted and what an 'appropriate amount' of 5% glucose was; estimates of the curvature of the optical fibers; a diagram/image of the pulley system used (as this is somewhat more of a methods paper); information about the power of light used and the duration of light delivery; how long it took to detect a SWR and intervene (especially relative to the total duration of a SWR)

- Data to help the reader assess the data, including tissue processing showing correct targeting of the optical fibers and tetrodes; examples of recordings including examples of single units that remained stable over the 9 hours of recording; examples of detected SWRs that were and were not interrupted; data associated with the successful inhibition of putative excitatory units (data was collected and discussed in the discussion section, but not presented in the results and never shown) and a lack of inhibition of inhibitory units (if the authors want to make the statement that they are not inhibited); information about the number of SWR detected, and the number of false positives and false negatives; how many cells per animal were included in the analysis and the general location of these cells including hemispheric information and rough septo-temporal (/anterior-posterior) location and putative cell type; per the methods section, tracking information is available, and if provided, would support that the weight of the system did not interfere too substantially with the animals movement across the board

- Analysis. The use of 60 channels should give the authors an impressive amount of data to examine, but the analysis of the data is currently rather limited. For example, the statement is made that “place maps... ...were destabilized”, but place maps aren't looked at.

- Statistics.

In Fig 7E and 7F, the data points are not independent of each other. By using each individual reward position as an individual observation, this violates the assumption of independence of observations in the Mann-Whitney test. Data should be considered at the mouse, not well, level.

It is unclear how data from different animals was handled in data analysis associated with Figure 8.

MINOR

- Some measure of variability should be including in Fig7A-D

- A brief explanation for only including female mice should be included.

- More information about the pre-training criterions and time (and number of trials) to reach them should be included

- The “sleep” periods may be better described as “rest” periods

- There is no need to have an entire figure to explain the cross of mouse lines used.

- It is unclear why a high magnification image of expression in the dentate, rather than the CA1, is shown in Fig2.

-Authors' names are listed on the title page.

-The authors state that their analysis requires that “all cells be inhibited to exactly the same extent”. This is not going to be achieved with their methods, as different amounts of light will reach different cells.

-Figure 2 is not referenced in the main text.

-From the discussion section it appears that multiple 9hr recording sessions may have been done in the same animal - is this interpretation of “our tetrodes were always adjusted in the evenings after the completion of the recordings” accurate? If so, what data is included/not included in the manuscript for each animal?

Comments by R2:

Guerrero et al provide a detailed description of a novel robust experimental preparation for highly parallel recording of neuronal activity and optogenetic inhibition in the hippocampus of behaving mice. They address mouse genetics, engineering, behavioural and data analytical aspects of the method in very detail. They further apply this technique to inhibit sharp-wave ripples during sleep and to study their relevance for the experience-dependent stabilization of a CA1 population firing. These novel findings extend earlier work of authors with recordings in a new mouse learning paradigm and provide an important replication of an earlier report (van de Ven et al, 2016) - performed here with internal controls. Guerrero et alsubstantially advance currently available tools for manipulation of ensemble activity in the hippocampus and potentially other brain regions. I have several comments aiming at further increasing applicability of the protocol.

What happens with LFP SPWs or neuronal firing during SPWs during closed-loop optogenetic inhibition? This information is essential since the manuscript is focused on SPWs.

136-138 Details about dura mater removal (if not removed with the bone) and discussing impact of local bleeding would be helpful.

149-151 are confusing - should not wax protect brain surface from drying and thus immobilizing electrodes?

238-239 What power of light was applied and how quickly was it reached upon the SPW detection/laser activation? Long-wave lasers may require longer time to achieve sufficiently high-power output.

Please define illumination conditions during the task.

Figure 1 Perhaps it is appropriate to code the whole Arch-EGFP-ER2 green since ER2 is also translated.

Figure 2 It is a bit difficult to estimate patterns of somatic expression and state plasma membrane localization based on the image - the dendritic signal is too strong for an adequate contrast in str. pyramidale.

286 Figure 2 instead of Figure 1
